# Modelling the direct virus exposure risk associated with respiratory events

**DOI:** 10.1098/rsif.2021.0819

**Published:** 2022-01-12

**Authors:** Jietuo Wang, Federico Dalla Barba, Alessio Roccon, Gaetano Sardina, Alfredo Soldati, Francesco Picano

**Affiliations:** ^1^ Centro di Ateneo di Studi e Attività Spaziali - CISAS, University of Padova, Padova 35131, Italy; ^2^ Department of Industrial Engineering, University of Padova, Padova 35131, Italy; ^3^ Institute of Fluid Mechanics and Heat Transfer, TU Wien, Vienna 1060, Austria; ^4^ Polytechnic Department, University of Udine, 33100 Udine, Italy; ^5^ Department of Mechanics and Maritime Sciences, Chalmers University of Technology, 41296 Gothenburg, Sweden

**Keywords:** infectious diseases, virus transmission, low-order model, airborne transmission

## Abstract

The outbreak of the COVID-19 pandemic highlighted the importance of accurately modelling the pathogen transmission via droplets and aerosols emitted while speaking, coughing and sneezing. In this work, we present an effective model for assessing the direct contagion risk associated with these pathogen-laden droplets. In particular, using the most recent studies on multi-phase flow physics, we develop an effective yet simple framework capable of predicting the infection risk associated with different respiratory activities in different ambient conditions. We start by describing the mathematical framework and benchmarking the model predictions against well-assessed literature results. Then, we provide a systematic assessment of the effects of physical distancing and face coverings on the direct infection risk. The present results indicate that the risk of infection is vastly impacted by the ambient conditions and the type of respiratory activity, suggesting the non-existence of a universal safe distance. Meanwhile, wearing face masks provides excellent protection, effectively limiting the transmission of pathogens even at short physical distances, i.e. 1 m.

## 1. Introduction

Since the coronavirus disease 2019 (COVID-19) pandemic outbreak in the spring of 2020, extensive studies have been carried out to significantly advance our understanding of different scientific problems, ranging from the transmission pathways of respiratory diseases to mitigation strategies for reducing the infection risk [1]. The severe acute respiratory syndrome coronavirus 2 (SARS-CoV-2) virus, like many other respiratory viruses, spreads from an infected individual to other susceptible individuals through virus-laden droplets emitted while breathing, speaking, singing, coughing and sneezing. These droplets span a wide range of diameters: from large drops, O(1 mm), to small droplets, O(1 μm) [2–6]. These droplets, released together with a puff/jet of buoyant moist turbulent air, are then dispersed in the surrounding ambient air and exposure or inhalation of these droplets leads to a potential risk of infection [6–8]. Investigating the transport and deposition of these droplets is thus of fundamental importance to design effective guidelines for disease transmission prevention.

The transport and deposition of respiratory droplets is controlled by the competition among gravitational forces, drag forces and evaporation. Depending on the outcome of this competition, we can distinguish among three infection routes. The first route can be identified in the fomites: droplets that settle along their trajectories can contaminate surfaces, and a susceptible individual, touching the contaminated surface, can get infected [9,10]. The other two possible routes of infection can be linked to the inhalation and subsequent deposition of pathogen-laden droplets in the respiratory mucosa: droplet direct transmission and airborne transmission. The key differentiating factor between these two routes is the aerodynamics behaviour of the pathogen-laden droplets. Droplet transmission [11] refers to the infection propagation associated with large droplets that behave ballistically, the motion of which is governed by gravity. In contrast, airborne transmission [12–14] refers to the spread of the disease associated with smaller droplets that behave as aerosols, the motion of which is governed by aerodynamic drag and evaporation, i.e. droplets/particles that remain airborne and can spread for long distances.

As the latest research suggests that fomites are unlikely to be a major source of infection for SARS-CoV-2 [12,15,16], understanding the role played by the two latter infection routes is crucial to establish effective guidelines for pathogen transmission prevention. One of the first seminal works in this direction was written by Wells [17], who hypothesized that the interplay of evaporation and gravity determines the lifetime of respiratory droplets. In the picture described by Wells, there is a critical size (about 100 μm) for each specific environmental condition that dichotomizes particles that would fall to the ground and droplets that would evaporate out at the same time. Wells' theory is pioneering in differentiating droplet and airborne transmission, and is corroborated by the fact that small droplets could evolve into droplet nuclei by evaporation [18], forming micro-metric particles of non-volatile materials (e.g. mucus) that can potentially carry the virus and contributing to the spread of the disease. This framework is so concise, readable and expressive that, since reported, it has been largely used in most public health guidelines [5,8]. The Wells model, however, presents a major weakness [3,5,8,19–21]: the evaporation time of the droplets is estimated using the classical *D*^2^-law [22] (or constant temperature model), which states that the droplet surface reduces linearly over time at a rate determined by ambient conditions. This evaporation model ignores the presence of a turbulent cloud of moist air, which, as demonstrated in recent studies, plays a crucial role in the fate of respiratory droplets [1,23], as well as the presence of the surrounding droplets. This leads to incorrect estimates of the evaporation times and results in much shorter predicted droplet lifetimes [20,21].

To overcome these limitations, Xie *et al.* [3] developed an improved model capable of describing the evaporation and motion of droplets exhaled during respiratory events under different conditions (relative humidity (RH), air velocity and respiratory jets). The model consists of a detailed mathematical framework that describes droplet motion using nonlinear differential equations coupled with a low-order description of the buoyant turbulent puff. The model developed by Xie *et al.* [3], although more solid and accurate than Wells' theory, inevitably loses the conciseness advantage of the latter. With the aim of developing an effective yet simple model able to accurately predict droplet evaporation times, it is worthwhile noticing that an interesting picture emerges from recent studies on respiratory droplet evaporation [19–21]: although the resulting droplet lifetimes are much larger than classical *D*^2^-law predictions, the *D*^2^-law scaling seems still to bear some universality [21,24]. Specifically, the mean evaporation times seem to follow a *D*^2^-law-like scaling but with a different pre-factor. Inspired by this observation, a revised version of the *D*^2^-law has been recently proposed by Dalla Barba *et al.* [25]: using a proper estimate of the asymptotic droplet temperature, the evaporation rate of dilute droplets in sprays, jets and puffs can be accurately determined. Even if more accurate than the classical formulation, it should be remarked that the performance of the revised *D*^2^-law is affected by low-temperature and high RH environmental conditions. In particular, the non-monotonic time behaviour of the droplet surface, reported in Ng *et al.* [20] for these extreme conditions, cannot be fully captured even by the revised model.

In light of the most recent understanding of respiratory events, we use this body of developed knowledge to move away from isolated droplet emission to the turbulent, multi-phase puff model [1,5,7]. In this paper, we establish a comprehensive theoretical framework capable of accurately describing the evaporation and dispersion behaviour of pathogen-laden droplets emitted during expiratory events. The model revises the outdated Wells theory with the most recent knowledge on turbulent droplet transport by jets or plumes, as well as the state-of-the-art understanding of respiratory activities. The proposed model, although mathematically simple, provides a general assessment of the direct contagion risk during different respiratory activities and ambient conditions.

We start by assessing the accuracy of the revised *D*^2^-law [25] in evaluating the evaporation of respiratory droplets. Building on this, we propose a revision of the traditional Wells theory, including an effective correction to the classical Stokes drag needed for relatively large droplets. We show that the proposed model can accurately predict the evaporation–falling curve of droplets when compared with previous reference data [3]. Coupling the latest research on turbulent jets and puffs [3,7,21,26–29], we proceed by proposing an integrated framework able to describe the evaporation–falling dynamics of respiratory droplets for different environmental conditions and respiratory activities. Then, using the theoretical model and available data on respiratory events (e.g. droplet initial size distribution [2,5,21], viral load measurements [30,31]), we quantify direct virus exposure as a function of either physical distancing or face covering. Descriptions of more complex phenomena, e.g. droplet condensation [19–21] and the upward movement of buoyant puffs [3,7,8,21], are not considered in the proposed model, but their importance is critical for an indirect airborne contagion and would be our future focus.

## 2. Methodology and validation

**Figure 1.**
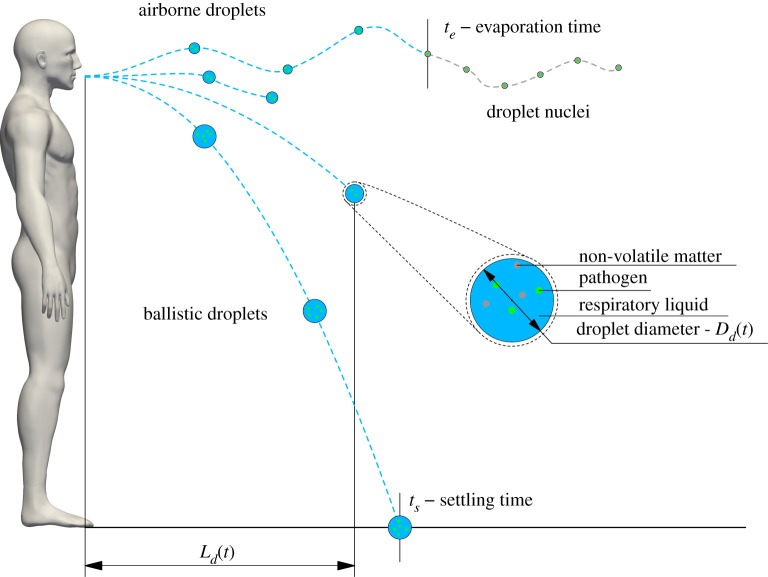
Sketch showing the main parameters of the model. For large ballistic droplets, the droplet lifetime is the settling time, *t*_*s*_, while for smaller airborne droplets, the droplet lifetime is the evaporation time, *t*_*e*_. The horizontal distance travelled by the respiratory droplets, *L*_*d*_(*t*), is also shown. The close-up view shows the droplet dimension and highlights the presence of pathogens and other non-volatile components in the respiratory liquid. For visualization purposes, the sketch is not to scale.

To evaluate the lifetime of respiratory droplets, *t*_*l*_, our model considers both the evaporation and settling dynamics of the droplets. Once the droplet lifetime is evaluated, the horizontal distance travelled by the droplets, *L*_*d*_, can also be calculated. A graphical representation of these model parameters is provided in figure 1. From a mathematical point of view, the model formulation can be summarized as follows:

2.1 $$t_{l}=\min(t_{e},t_{s}),$$

2.2 $$t_{e}=\frac{D_{d,0}^{2}}{k},$$

2.3 $$t_{s}=\frac{D_{d,0}^{2}-\sqrt{D_{d,0}^{4}-9\frac{4k\mu }{(\rho_{d}-\rho )gf}H_{d}}}{k},$$

2.4 $$\begin{array}{rl} L_{d} & =\left\{ \begin{array}{cc} L_{d,1}=\sqrt{4BU_{0}R_{0}}t_{l}^{1/2} & \text{when}\;t_{l}\leq t_{\mathrm{inj}},\\ L_{d,2}=\sqrt{4BU_{0}R_{0}}t_{\mathrm{inj}}^{1/4}t_{l}^{1/4} & \text{when}\;t_{l}>t_{\mathrm{inj}}, \end{array}\right. \end{array}$$

where *μ*, *ρ* and *ρ*_*d*_ are the dynamic viscosity and density of the ambient air and the droplet density, respectively, with *k* being the surface evaporation rate estimated by the thermodynamic properties of the droplet and environment [25]. The inertial Reynolds number of droplets with initial diameter $D_{d,0}^{2}$ is taken into account for the Stokes law with a fixed drag correction factor *f*. The initial height of the droplets away from the ground is defined as *H*_*d*_ and *U*_0_ is the velocity of a respiratory puff ejected from a mouth with characteristic size *R*_0_. *g* represents gravitational acceleration and *B* is the universal jet velocity-decay constant [32]. The lifetime of a respiratory droplet, *t*_*l*_, is determined by equation (2.1). Following the Wells formalism, a respiratory droplet can either evaporate or settle on the ground. Hence, the lifetime *t*_*l*_ corresponds to the minimum between the evaporation time, *t*_*e*_, expressed by equation (2.2), and the settling time, *t*_*s*_, determined by equation (2.3). Given the droplet lifetime, it is possible to predict the horizontal distance travelled by the droplet. This length is defined by *L*_*d*,1_ when the droplet lifetime is smaller than *t*_inj_, where *t*_inj_ identifies the duration of the jet phase of the respiratory event (i.e. the time during which momentum is injected in the ambient air). In contrast, if the droplet lifetime is larger than *t*_inj_, the horizontal distance travelled by the droplet is determined by *L*_*d*,2_. Indeed, once the jet phase is terminated, the flow behaves as a turbulent puff [23,29].

In the following, a brief description of each sub-part of the model is presented; additional details are provided in the electronic supplementary material. Equation (2.2) describes the evaporation time, *t*_*e*_, necessary for a droplet to dry out by assuming a linear reduction in time of its surface and a constant droplet temperature. The variable *D*_*d*,0_ represents the initial droplet diameter, whereas *k* is the surface evaporation rate estimated by the thermodynamic properties of the droplet and environment (see also the electronic supplementary material for details). A similar evaporation model, the so-called $D^{2}$-law, was introduced by Langumir’s pioneering work [22] and then adopted in the Wells theory [17]. Recently, it has been revised by Dalla Barba *et al.* [25] for the determination of *k,* where the asymptotic evaporation temperature of isolated, evaporating droplets is used instead of the initial one to calculate the evaporation rate. This revised $D^{2}$-law provides more accurate predictions for the evaporation of dilute droplets (see electronic supplementary material for details and validations against experimental data); for additional details, readers are referred to Dalla Barba *et al.* [25].

**Figure 2.**
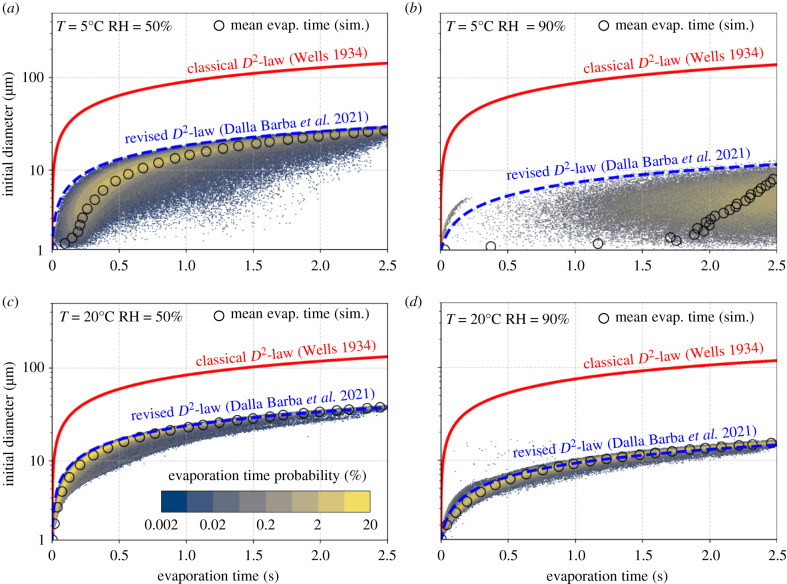
Comparisons of the evaporation time, *t*_*e*_, of water droplets of different initial diameters estimated by different models: Wells' theory [17] (red line), revised $D^{2}$-law [25] (blue line) and high-fidelity simulations of sneezing jet [21] (open black circles). For the high-fidelity simulations, in addition to the mean evaporation time (black circles), the probability density function of the evaporation times for any given initial diameter is shown with a blue–yellow map. Each panel refers to a different combination of temperature and RH values: $T=5^{\circ }$C and RH=50% and 90% (*a*,*b*), $T=20^{\circ }$C and RH=50% and 90% (*c*,*d*).

To further assess the accuracy of the revised $D^{2}$-law in predicting the evaporation times for respiratory droplets, we compare in figure 2 the predictions of the evaporation time of water droplets obtained from three different models: (i) the original Wells theory; (ii) the revised $D^{2}$-law evaporation model; (iii) the evaporation times obtained from high-fidelity simulations of sneezing events performed using a set-up similar to that adopted by Wang *et al.* [21]. Each of the four plots refers to a different combination of temperature and RH values. In each sub-figure, for a given initial diameter *D*_*d*,0_, red solid lines identify the predictions based on the Wells theory (classical $D^{2}$-law), i.e. assuming that the droplet temperature is constant and equal to the initial jet temperature. Likewise, blue dashed lines identify the predictions obtained from the revised $D^{2}$-law with the decay constant computed from the ambient temperature. Meanwhile, for the high-fidelity simulations, for any given initial diameter *D*_*d*,0_ the mean evaporation time is shown with open black circles. A blue–yellow colour map shows the probability of obtaining a certain evaporation time.

The revised $D^{2}$-law is capable of predicting the mean evaporation behaviours of respiratory droplets accurately. However, for the low-temperature and high-RH case, e.g. $T=5^{\circ }$C RH=90% (figure 2*b*), good results are observed only for long times, i.e. *t* > 2.5 s. In low-temperature and high-RH conditions, where the emitted respiratory jet strongly differs from the ambient air, the local micro-environments are critical in determining the temporal evolution of the droplet dynamics [21], which differs from a linear decay of the droplet surface. Ng *et al.* [20] observed that, under these conditions, air could not contain much moisture. Therefore, the warm and humid exhaled vapour puff becomes supersaturated when entering the cold ambient air, so the droplets carried by the puff tend to experience vapour condensation before evaporating at long time. The colder the ambient air, the more important this non-monotonic behaviour is [20,25]. To improve the accuracy of the evaporation model, especially for short time evolution, we propose here a minor improvement to the revised $D^{2}$-law. In particular, as reported in Dalla Barba *et al.* [25], a small transient time has been observed for the droplet temperature in order to reach the asymptotic value starting from its initial one. This transient time has been observed to be a few droplet relaxation times, $\tau_{d,0}=\rho_{d}D_{d,0}^{2}/18\mu$, corresponding to the typical inertial time scale of the droplet in a viscous flow (*ρ*_*d*_ is the droplet density and *μ* is the air viscosity). To account for this, we introduce a two-stage evaporation model: in the initial step of the evaporation process, i.e. *t* < 6*τ*_*d*,0_, the droplets evaporate with a rate *k* determined by the initial droplet temperature, as in the classical $D^{2}$-law; then, the evaporation rate *k* is computed using the asymptotic droplet temperature, as in the revised $D^{2}$-law (see electronic supplementary material for details).

Moving to the settling dynamic, equation (2.3) predicts the settling time *t*_*s*_, i.e. the time needed to reach the ground from a specific falling height *H*_*d*_, here fixed to 2 m for consistency with previous studies [3,17]. In the Wells theory, the settling time is estimated using the Stokes drag law, which holds for tiny droplets at small droplet Reynolds number *Re*_*d*_ = *ρu*_*s*_ *D*_*d*_/*μ* ≪ 1, i.e. in the viscous dominated regime, with *ρ* and *μ* being the density and dynamic viscosity of the air, respectively. This hypothesis is realistic for small respiratory droplets whose Reynolds number, *Re*_*d*_, is supposed to be sub-unitary. However, for large droplets (200 μm or greater), the Reynolds number based on settling speed could be around 10 or more. As reported in Seinfeld & Pandis [33], the Stokes law may overestimate the settling speed by 60% for these large droplets. To account for finite (small) Reynolds number effects, we propose a correction to the Stokes law by a fixed drag correction factor *f*, which we show provides good predictions for respiratory droplets (see electronic supplementary material for details). In particular, to avoid a nonlinear model, we define the drag correction factor using an average constant droplet Reynolds number $Re_{d}^{*}=\rho u_{s}D_{d}^{*}/\mu$, where $D_{d}^{*}$ is an average droplet diameter and $u_{s}=(\rho_{d}-\rho )g{D_{d}^{*}}^{2}/18/\mu$ is the Stokes terminal velocity,

2.5 $$f=\frac{1}{1+0.15{Re_{d}^{*}}^{0.687}};$$

2.6 $$Re_{d}^{*}=\frac{1}{18}\frac{\rho_{d}-\rho }{\mu^{2}}g\rho {D_{d}^{*}}^{3};$$

**Figure 3.**
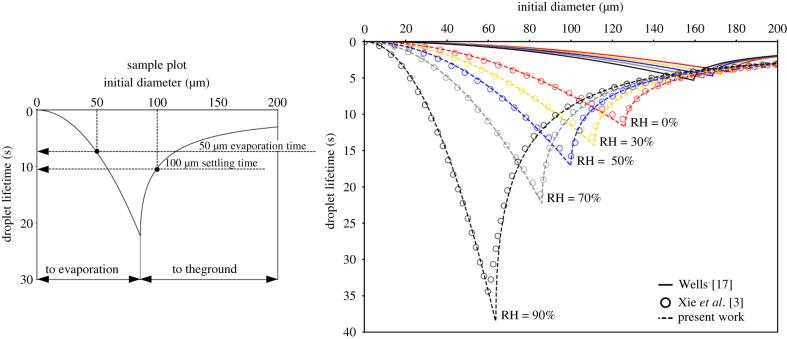
The right panel shows the comparison of the predicted droplet lifetime of water droplets having different diameters obtained from different models: Wells [17] (solid lines), Xie *et al.* [3] (symbols) and the present model (dashed line). The sample plot on the left guides the reading of the main panel: for small droplet diameters, the lifetime is determined by evaporation (left branch of the plot; to evaporation), while, for larger droplet diameters, the lifetime is determined by the settling dynamics (right branch of the plot; to the ground). The plot refers to an ambient temperature equal to $T=18^{\circ }$C and different RH values are considered: RH=90% (black), 70% (grey), 50% (blue), 30% (yellow), 0% (red).

2.7 $${D_{d}^{*}}^{3}=\frac{D_{d,0}^{3}+D_{d,t}^{3}}{2}≃\frac{D_{d,0}^{3}}{2};$$

where *D*_*d*,0_ and *D*_*d*,*t*_ are the initial and terminal droplet diameters, respectively. Figure 3 compares the droplet lifetime predicted by the Wells theory [17] (continuous line) and the model proposed here (dashed lines) against the model [3] (empty circles) for different droplet initial diameters. As highlighted from the sample plot reported on the left, the droplet lifetime is the minimum between the evaporation time (lifetime for small droplets) and the falling time (lifetime for large droplets), as also illustrated in equation (2.1). Colours are used to distinguish among the different humidity values considered: RH=90% (black), 70% (grey), 50% (blue), 30%(yellow), 0% (red). The initial temperature of droplets and ambient air are *T*_*d*,0_ = 33°C and *T* = 18°C, respectively. It is worthwhile noting that, in Xie *et al.* [3], a detailed (Lagrangian) description of the droplet dynamics was employed together with a (Eulerian) model for the spatial–temporal evolution of respiratory jets. The complete model results in a system of nonlinear differential equations that must be numerically integrated for any fixed environmental condition. Hence, this complex framework produces accurate data that can be used as a benchmark. From figure 3, we can observe how the Wells model largely underestimates the droplet lifetime even by more than one order of magnitude for middle-size droplets (upper right part of the plot). On the contrary, the algebraic model proposed here can accurately capture the droplet lifetime in both the evaporating (left, *t*_*e*_) and falling (right, *t*_*s*_) branches of the plot for all ambient conditions considered. In addition, the present results are in excellent agreement with the reference data of Xie *et al.* [3].

Using this simple and effective model, it is also possible to estimate the horizontal distances travelled by the respiratory droplets during different respiratory events. Indeed, all the different respiratory flows (e.g. speaking, coughing and sneezing) can be modelled as a jet phase, during which the exhaled gas is emitted, followed by a puff phase [5,7,23]. On these grounds, equation (2.4) determines the horizontal travelling distance of respiratory droplets, where *B* ≃ 6, *U*_0_ and *R*_0_ indicate the universal jet velocity-decay constant [32], the ejection velocity and the inlet radius (i.e. a characteristic size of the mouth), respectively. Equation (2.4) has been derived assuming that the droplet velocity is equal to the gas-phase velocity in the geometric centre of the inlet and assuming a two-phase propagation process of the expelled air. In particular, we consider a turbulent jet phase having duration *t*_inj_, followed closely by a puff phase after the end of the ejection phase. For details on the two-phase propagation theory of turbulent puffs, readers are referred to the following references: Sangras *et al.* [26], Xie *et al.* [3], Bourouiba *et al.* [7], Wei & Li [27], Abkarian *et al.* [28] and Wang *et al.* [21].

## 3. Results

**Figure 4.**
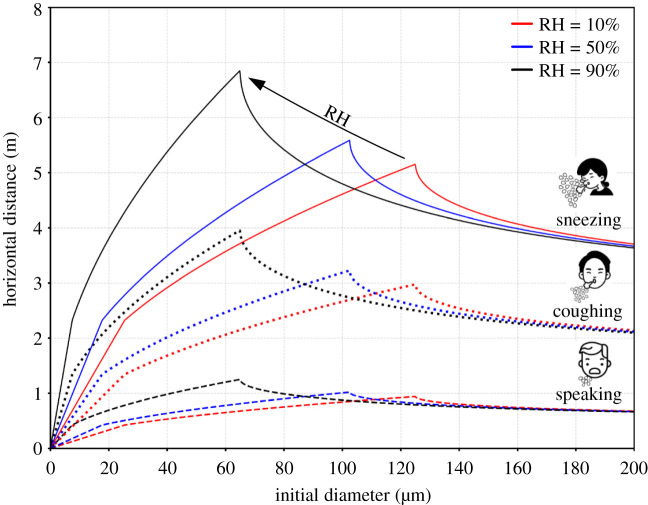
Comparison of the predicted horizontal distances travelled by respiratory droplets, *L*_*d*_, for different ambient conditions: RH=10% (red), RH=50% (blue) and RH=90% (black) as a function of the initial droplet diameter. Each respiratory event is reported with a different line style: speaking (dashed), coughing (dotted) and sneezing (solid). The present results are obtained considering an ambient temperature of $T=20^{\circ }$C and an initial droplet temperature of $T_{d,0}=35^{\circ }$C. The characteristic velocity of the different respiratory events is assumed equal to: 1 m s^−1^ (speaking), 10 m s^−1^ (coughing) and 30 m s^−1^ (sneezing).

Using the proposed model and considering different respiratory events [3,34,35], we can estimate the horizontal distance travelled by the droplets as a function of the initial diameter for different ambient conditions, as illustrated in figure 4. We can observe that droplets emitted during different respiratory events reach different distances before evaporating or falling depending on the ambient RH (RH=10% – red, RH=50% – blue and RH=90% – black). In particular, considering the most violent respiratory act (sneeze), 60 μm droplets can reach a distance of nearly 7 m. This prediction is consistent with the experimental findings reported in Bourouiba [6,36]. Droplets of a similar size, emitted when speaking, can reach a distance slightly longer than 1 m, in agreement with Abkarian *et al.* [28]. Reducing the RH, droplets with a larger initial size reach shorter maximum distances: the evaporation dynamics becomes faster and for 60–100 μm droplets the lifetime decreases. Overall, our model estimates indicate that respiratory droplets can reach a maximum distance between 5 and 7 m when sneezing, 3–4 m when coughing and ≃1 m when speaking.

Once the distance travelled by the respiratory droplets and their lifetime are known, assuming an initial droplet size distribution (e.g. a lognormal distribution [5,21]), and a certain viral load, we can estimate the virus exposure. As viral load is characterized by large uncertainty [30,31,37–41] and exhibits strong variations during the different infection stages [30,31,42], we compute the exposure in a dimensionless form [21]. The dimensionless virus exposure is defined as the ratio of the cumulative initial volume of droplets survived at distance *L*_*d*_ to the total volume of droplets at the beginning. This definition is based on three hypotheses: (i) a uniform viral load across all droplets, i.e. the virus copies inside each droplet are directly proportional to the droplet initial volume; (ii) no viral load decay, i.e. the number of virus copies is fixed by the initial droplet size; (iii) the virus exposure becomes null when a droplet reaches the ground or completely evaporates. By assuming that all respiratory events are initially characterized by the same droplet size distribution, we provide virus exposure maps normalized by the total amount of virus copies emitted during the most violent respiratory event (sneezing). Since each respiratory event has been experimentally characterized by a different average droplet number [2], i.e. 1 000000, 5000 and 250 for sneezing, coughing and speaking, respectively, the initial number of emitted virus copies is different, and so is the virus exposure at time *t*. However, it is worth noting that the droplet number for speaking obtained from Duguid experiments [2] does not correspond to a single event but counts during ‘speaking loudly one hundred words’, since its definition is not as distinct as coughing or sneezing events. Chao *et al.* [43] estimated that the total number of droplets expelled during speaking ranged from 112 to 6720 by counting 1–100 numbers. Asadi *et al.* [44] reported that the droplet emission rate during normal speech is positively correlated with the loudness of vocalization, ranging from approximately 1 to 50 s^−1^ particles for low to high amplitudes, regardless of the language spoken. However, more recent studies [45,46], by visualizing small speech droplets with an intense sheet of laser light, revealed mean droplet emission rates of 1000 s^−1^ with peak emission rates as high as 10 000 s^−1^, indicating a higher total integrated volume than previous results [2,4,43,44]. Overall, the droplet number employed here to compute the initial virus exposure related to speaking events could be a conservative estimate, but is at least a reasonable value.

**Figure 5.**
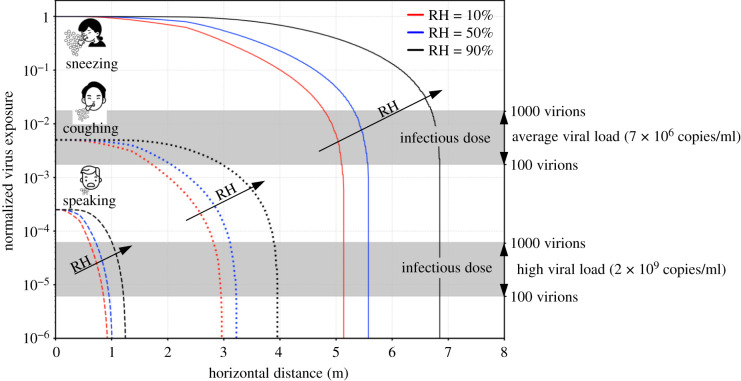
Normalized virus exposure as a function of the horizontal distance from an infected individual for different ambient conditions: RH=10% (red), RH=50% (blue) and RH=90% (black). Three distinct respiratory events are considered with the same initial droplet size distribution, i.e. a lognormal distribution with $\hat{\mu }=\ln(12\;\mu m)$ and $\hat{\sigma }=0.7$ [5]. Present results are obtained considering an ambient temperature of $T=20^{\circ }$C and an initial droplet temperature of $T_{d,0}=35^{\circ }$C. The characteristic velocity of the respiratory events considered is assumed equal to 1 m s^−1^ (speaking), 10 m s^−1^ (coughing) and 30 m s^−1^ (sneezing). As a reference, the infectious dose (which ranges from 100 to 1000 virions [47,48]) is reported with two grey bands for two viral loads: an average case (7 × 10^6^ copies ml^−1^) [30,40] and an extreme case (2 × 10^9^ copies ml^−1^) [30].

Using these assumptions, we show in figure 5 the spatial evolution of the virus exposure for different respiratory events and ambient conditions: RH=10% (red), RH=50% (blue) and RH=90% (black). An important picture emerges from the virus exposure maps: a well-defined *safe distance* is not only related to the respiratory events but also determined by the ambient conditions. These results imply that it is impossible to define a universal standard safe distance. In particular, the safe distance should be extended when the ambient relative humidity is high. A 1 m social distance might be enough when people talk, but not when coughing, especially in high RH conditions. It is also true that coughs and sneezes are less frequent than standard talking among groups of people, so policymakers may consider this aspect to evaluate a global infection risk. We believe that, rather than a single safe distance, it is possible to evaluate a quantitative reduction in the infection risk as a function of the social distance and different RH values. Policymakers may exploit these maps to determine the impact of a different social distance prescription on reducing the direct infection risk. To also provide an indication of the infection risk associated with the different respiratory events, the infectious dose for SARS-CoV-2 (from 100 to 1000 virions [47,48]) is reported with a grey band for two different viral load values: an average case (7 × 10^6^ copies ml^−1^) [30,40] and an extreme case (2 × 10^9^ copies ml^−1^) [30] (given the droplet number and distribution in a respiratory event, the overall liquid emitted is fixed, e.g. 0.0081 ml for a sneeze). An exhaustive discussion on the infection risk in relation to these thresholds is however difficult: viral loads (and thus the position of these bands) are characterized by a large variability and depend on many different factors (e.g. disease severity, infection stage, vaccination status, etc.). From the present model, it appears that, in the case of a high viral load, it is possible to be directly infected when talking at a 1 m distance for around 1 min. In addition, it should noted that overall virus exposure depends also on the indirect transmission route and so on the exposure time (i.e. the overall time in the proximity of an infected individual, especially in closed environments).

The use of face masks against SARS-CoV-2 transmissions has substantially increased since the outbreak of the COVID-19 pandemic, although significant controversy on their effectiveness existed and recommendations on this measurement varied across countries [49]. More recently, wearing face masks has been widely accepted as a well-established protective measurement, providing both ‘inward’ protection by filtering virus-laden aerosolized particles that would be inhaled by an uninfected person and ‘outward’ protection by trapping virus-laden droplets expelled by an infected person [1]. Recent studies [21,50–54] have provided evidence that supports the ability of face masks in reducing pathogen transmission. We focus here on the ‘outward’ protection provided by two standard face coverings, i.e. surgical and N95 masks, and take into account two effects: a lower jet velocity and a trapping of virus-laden droplets. The first aspect is implemented by simply assuming a reduction of the emitted flow velocity induced by the mask presence, which is estimated from the experimental data reported in Prasanna Simha & Mohan Rao [55]. In particular, we assume that the velocity of the expelled puffs reduces to one-quarter of its *original* value when using a surgical mask and one-eighth for an N95 mask. The second aspect is accounted for by following the study by Cheng *et al.* [53], in which the size-dependent particle penetration rate of surgical and N95 masks based on the past literature [56,57] is calculated. Additional findings on face mask efficiency can be found in the experimental study of Bagheri *et al.* [54] and in the theoretical one of Mao & Hosoi [58], with the latter mainly focused on airborne aerosols with size smaller than 1 μm. As it is known that the breathing airflow could escape from the edge area owing to improper mask fit [21,51], for each type of mask, we also consider a case in which leakages of respiratory droplets occur. Using the data provided in Cheng *et al.* [53], we treat leakage as reduced efficiency in the size-dependent droplet mask filtering. In other words, we consider the leakage as a higher number of droplets passing through the mask. Although not perfect, this model assumes the worst-case scenario where the leaked droplets are totally carried by the jet flow.

**Figure 6.**
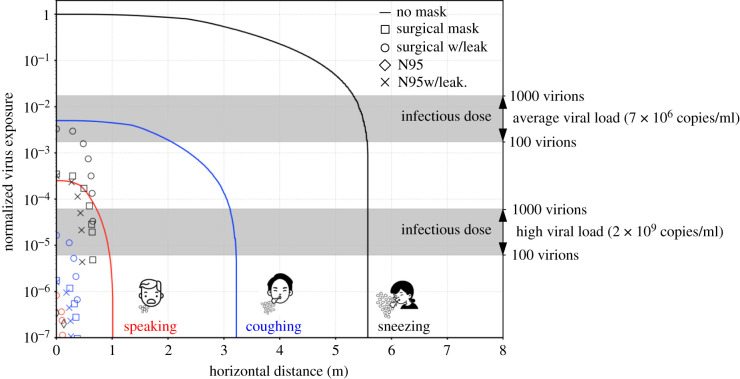
Normalized virus exposure maps for different face coverings: no mask (solid line), surgical mask (open squares), surgical mask with leakage (open circles), N95 mask (crosses), N95 mask with leakage (open diamonds). Colours are used to distinguish among the different respiratory events: sneezing (black), coughing (red) and speaking (red). We assume that the puff velocity decays to one-quarter (or one-eighth) of its initial value after passing a surgical mask (or N95 mask) [55]. For perspicuity reasons, only one ambient condition ($T=20^{\circ }$C and RH=50%) is considered. As a reference, the infectious dose (from 100 to 1000 virions [47,48]) is reported with a grey band for two viral load values: an average case (7 × 10^6^ copies ml^−1^) [30,40] and an extreme case (2 × 10^9^ copies ml^−1^) [30].

Figure 6 shows the normalized virus exposure obtained with different types of face covering: no mask (solid line), surgical mask (open squares), surgical mask with leakage (open circles), N95 mask (crosses), N95 mask with leakage (open diamonds). Colours are used to identify the different respiratory events: speaking (red), coughing (blue) and sneezing (black). For simplicity, only one ambient condition is considered: $T=20^{\circ }$C and RH=50%. From figure 6, it appears that wearing masks is a very effective measure in reducing virus exposure and N95 masks show a superior protection performance. In particular, at close distances, the virus exposure associated with an infected person wearing a surgical mask is about three orders of magnitude lower than its corresponding value without the mask. Likewise, properly wearing an N95 mask could further decrease the exposure to an almost vanishing level of infection, i.e. ~*O*(10^−6^). This observation can be traced back to the ability of masks in blocking large droplets, which load more virus copies than smaller droplets (assuming a uniform viral load across all droplets diameters). Indeed, experimental data recently published showed that masks are highly effective in blocking respiratory droplets larger than about 20 μm [53,54]. In addition, the suppressing effect of masks on airflow velocity results in a shorter propagation distance for droplets, effectively limiting the infection risk even near an infected person, i.e. 1 m. Considering both these mechanisms—the blocking effect of masks on respiratory droplets larger than 20 μm and the suppressing effect on airflow velocity—it is clear that wearing masks significantly reduces the spreading of infectious droplets and provides excellent ‘outward’ protection.

Concluding the discussion, the proposed model presents the great potential to predict the evaporation and dispersion of infectious droplets, leading to reasonable estimates of direct virus exposure. Hence, it could be considered a revision of the seminal Wells model that incorporates the latest knowledge on multiphase flow modelling applied to respiratory flows. In any case, considering the aim of the present study, this cannot be applied to estimate the indirect transmission route dominated by aerosolized droplets carried out by the environmental flows. On this point, several recent models have been proposed. For example, Yang *et al.* [59] incorporated the results of high-fidelity simulations on speech jets [28], and proposed a simplified dose–response model to quantify the spatio-temporal dependence of virus infection risk driven by speech-generated aerosols between two speakers. This model assumes the continuous speaking activities to be quasi-steady, jet-like flows and fast aerosolization of speech droplets. They showed that both physical distancing and exposure time should be imposed to control infection probability. In particular, speakers should keep the contact time below 8 min for physical distancing of 1 m and 16 min for 2 m. Recently, Bazant & Bush [60] developed a theoretical model of long-term airborne transmission considering the number of occupants and their time spent in an enclosed space. Their model adopts the well-mixed assumption and quantifies the extent to which infection risk could be impacted by practical parameters, such as ventilation, room volume, the penetration rate of face masks and disease transmission tolerance. They suggested that less vigorous respiratory activities, using face masks and large rooms with high air exchange rates could effectively mitigate the transmission risk related to aerosolized virions. Other models recently proposed for the indirect route can be found in [61–64]. The authors would like to note that the aforementioned models focus on indirect airborne virus transmission, whereas the mathematical framework presented in this paper focuses on direct exposure (short-range airborne and droplets). Hence, the present model is complementary to the other models since both transmission routes are concurrently active.

## 4. Conclusion

Droplets released during respiratory events play a crucial role in transmitting respiratory diseases (e.g. SARS-CoV-2) from an infected host to a susceptible individual. In this study, we developed a simple physical model to predict the evaporation–falling–travelling performance of droplets expelled during respiratory activities under different ambient conditions. The proposed model revises the outdated Wells theory by exploiting the better knowledge on turbulent droplets transport by jets or plumes developed in the last few decades, as well as the state-of-the-art understanding of respiratory ejections. The model relies on a simple algebraic formulation and, without the need to solve complex systems of nonlinear differential equations, it is capable of accurately estimating the dispersion, evaporation and settling behaviours of droplets within a turbulent multiphase jet/puff. The model takes into account different respiratory events (speaking, coughing, sneezing) and different ambient conditions (temperature and RH) except for very cold and humid environments where droplet surface time decay would not be simply characterized by a linear behaviour [20]. Reference data from a well-recognized model [3], high-fidelity simulations [20,21] and the latest experimental investigations [65] have been used to benchmark the present model. Using the proposed framework, we systematically assess the effects of physical distancing and face coverings on virus exposure maps and thus on the infection risk. We show that the infection risk is vastly impacted by the ambient conditions and respiratory event considered, indicating the non-existent of a universal safe distance. Finally, using the proposed model and exploiting experimental data on the penetration of respiratory droplets through face masks [53,55–57], we assess the effects of face masks on the infection risk. We confirm that face masks provide excellent ‘outward’ protection, effectively reducing the infection risk near an infected person. Overall, we believe that the present model represents a substantial improvement on older models [3,17] and, owing to its simple but effective mathematical background, it can be widely used by policymakers to design effective guidelines for the prevention of direct contagion.

## Data accessibility

The datasets analysed in the present study have been deposited in Figshare (doi:10.6084/m9.figshare.16826374). The data are provided in the electronic supplementary material [66].
